# Integrating epigenetic data into molecular casual networks

**DOI:** 10.1186/1744-8069-10-S1-O21

**Published:** 2014-12-15

**Authors:** Seungyeul Yoo, Eunjee Lee, Jun Zhu

**Affiliations:** 1 Department of Genetics and Genomic Sciences; Icahn Institute of Genomics and Multiscale Biology, Icahn School of Medicine at Mount Sinai, New York, NY 10029, USA

## 

Genome-wide association studies (GWAS) have recently identified many risk loci for complex human diseases. However, genetics can explain only a fraction of disease variation. Epigenetics refers to cellular mechanisms that affect gene expression without modifying DNA sequence[1]. Epigenetic mechanisms reflect gene X environment interactions, which contribute to risk for many chronic diseases including obesity [2], hypertension [3], cancers [4], chronic inflammation [5], chronic pain [6], and chronic obstructive pulmonary disease (COPD) [7]. While these studies have provided an initial look into genetic or epigenetic factors affecting disease risk or disease severity, understanding the transcriptional regulation by genetic and epigenetic factors, such as DNA methylation and microRNA, may shed light on understanding the biological processes and molecular mechanisms associated complex human diseases.

By integrating genetic, epigenetic, and transcriptomic data we developed genetic causality tests [8,9] and a novel methylation-based causality test. Then, we developed a method to construct a global Bayesian network [10-12] using the causal test results as priors. As a proof-of-concept, we applied these methods to genome-wide genetic, epigenetic, and transcriptomic data and phenotypic data generated from lung tissues of COPD patients and non-COPD controls, and identified multiple causal regulators for pathways associated with disease severity. We experimentally validated candidate genes in cell lines, mouse models, and in human tissues. Our results suggest that the integrative causal network can provide important insights into understanding the mechanisms underlying epigenetic regulations, altering transcriptional programs that lead to COPD pathogenesis and progression. These approaches can be applied to uncover molecular mechanisms underlying other diseases, such as chronic pain.
